# Integrating predictive models and GWAS to identify candidate loci and genes for agronomic traits in rice

**DOI:** 10.3389/fpls.2026.1942478

**Published:** 2026-08-21

**Authors:** Qiang Zhou, Fujuan Wang, Yanlin Yang, Liping Dong, Yongrun Cao, Shumei Li, Guofang Zhang

**Affiliations:** 1 College of Agriculture, Xinyang Agriculture and Forestry University, Xinyang, China; 2 Hainan Seed Industry Laboratory, Sanya, Hainan, China

**Keywords:** agronomic traits, GWAS, metabolites, predictive models, rice

## Abstract

Rice is a staple crop whose improvement relies on breeding advances; precision agriculture demands predictive loci/genes for agronomic traits to innovate rice production. To cut experimental costs and boost efficiency, this study built predictive models using seedling leaf metabolomes to forecast rice agronomic traits and decode trait correlations. We integrated 11 agronomic traits and 840 metabolites from 524 rice germplasms, plus 17 agronomic traits of 3,000 varieties. Five algorithms (RF, LightGBM, SWR, LASSO, CART) were combined to build multi-model prediction systems, offsetting defects of single models for accurate complex trait prediction. GWAS on predicted phenotypes detected nine genetic hotspots. Results showed LASSO and CART had weak generalization, while LightGBM, RF and SWR delivered trait-specific predictive performance. Seven candidate genes within hotspots were validated via variation annotation, haplotype and tissue expression analyses. Distinct from laborious, environment-prone traditional phenotyping, these models realize rapid, stable high-throughput trait prediction via early metabolic markers. The uncovered loci and genes lay groundwork for dissecting molecular regulatory networks linking rice agronomy and metabolism.

## Introduction

Rice, a staple food crop worldwide, faces increasing threats to its production due to the escalating global population and the shrinking arable land area with each passing year. The adverse impact on food security is becoming increasingly apparent (Li et al., 2006). To address this challenge, a promising approach is the utilization of advanced agricultural techniques, notably precision agriculture, to optimize rice yields. Simultaneously, efforts must be made to cultivate superior rice varieties that are highly resistant, high quality, and yield-enhancing. Fortunately, advancements in high-throughput sequencing technology have permitted the resequencing of numerous genomes, offering the potential for efficient genotyping and widespread genetic variation surveys (Huang et al., 2012). While breeders have made remarkable progress toward these goals, the genetic basis remains poorly understood, particularly the relationships among complex agronomic, metabolic, and agronomic-metabolic traits.

Rice yield is determined by three key components: panicle number per plant, grain number per panicle, and grain weight. These three traits interact coordinately to shape final grain yield. Future rice improvement requires knowledge-driven breeding and a deep understanding of gene functions, diversity, networks, and their associations with key traits (Yano et al., 2016). Yield-related traits are complex polygenic characteristics that hamper dissection of their genetic basis. To develop high-yielding, stress-tolerant, high-quality rice with stable production, breeders mainly rely on three core strategies: optimizing plant morphological architecture, exploiting heterosis, and pyramiding elite genes. With expanded germplasm and lower sequencing costs, GWAS, a time-saving, cost-effective tool, is increasingly used to identify candidate QTLs/genes for complex rice traits and parse their genetic basis (Wang et al., 2020). Previous genome-wide studies on rice morphological characteristics, flowering, yield, drought resistance, quality and metabolism have laid a solid foundation for breeding superior varieties (Huang et al., 2012; Chen et al., 2014; Dong et al., 2018; Guo et al., 2018; Zhou et al., 2020). Although these results provide valuable references for future breeding, few studies have integrated metabolites as predictive indicators together with complex agronomic traits for subsequent GWAS analysis.

Plant metabolites, crucial for growth, development, and environmental adaptation, play pivotal roles in biological processes (Yang et al., 2021). Alkaloids are bitter and deter herbivores. Polyphenols are plant secondary metabolites with antioxidant activity that scavenge free radicals and protect cells (Obuekwe et al., 2020; Vassiliadis et al., 2023). Metabolomics is a powerful tool for identifying metabolic genes and constructing networks, crucial for improving crop quality and yield, and promoting agricultural sustainability and food security (Luo, 2015). High-throughput metabolite profiling has been widely used to reveal metabolic diversity and its complex links to agronomic traits in rice (Chen et al., 2016; Hu et al., 2020). Modern high-throughput metabolite detection enables in-depth analysis of rice metabolic networks, revealing relationships between metabolites and agronomic traits. This advances rice biology and supports breeding for higher yield, resistance, and sustainability (Xu et al., 2016; Kang et al., 2019). The integration of multi-omics data is valuable for studying metabolic changes during crop domestication and stress responses (Zhu et al., 2018; Urrutia et al., 2021). Traditional metabolite studies are fragmented, ignoring agronomic traits and hindering understanding of trait-metabolite genetic interactions. The integration of metabolomics, pGWAS and mGWAS has revealed natural metabolic variation and its regulation in multiple crops (Chen et al., 2014; Wen et al., 2015; Zhu et al., 2018; Wang et al., 2018a; Chen et al., 2020). Previous rice GWAS on grain metabolism and phenotypes revealed shared genetic regulation across tissues/species, identifying candidate genes for grain color and size (Chen et al., 2016). These findings support metabolic-phenotypic associations; however, a global understanding of metabolic regulation remains limited (Tieman et al., 2017). Two milestone studies have laid an important theoretical foundation for using metabolic data to predict complex phenotypes of plants. The tomato research uncovered extensive genetic linkage between fruit metabolic loci and yield-related agronomic traits, demonstrating the feasibility of utilizing metabolite profiles to reflect crop quality and yield performance (Schauer et al., 2006). The subsequent study on *Arabidopsis* further confirmed that composite metabolic signatures, rather than individual metabolites, possess reliable predictive capacity for plant growth and biomass traits (Meyer et al., 2007). Several comprehensive reviews further consolidated this theory, emphasizing that metabolites function as intermediate biochemical phenotypes connecting genetic variation and final observable traits, and summarized the application prospects and bottlenecks of metabolome-based phenotypic prediction in diverse crops (Alseekh et al., 2021; Razzaq et al., 2022; Dantas et al., 2026). Accumulating studies have demonstrated the feasibility of constructing predictive models for rice agronomic traits based on metabolome data. Using metabolite profiles, researchers have successfully predicted a series of complex quantitative traits in rice, including grain yield, heading date, plant height, grain quality and yield heterosis, which provides an efficient strategy to accelerate rice breeding practice (Dan et al., 2016; Wei et al., 2018; Dan et al., 2019). These investigations highlight the great potential of metabolite-based prediction in dissecting phenotype-metabolite associations and guiding the selection of elite rice materials.

In this study, we performed GWAS on predicted agronomic values derived from predictive models, identifying nine reliable hotspots and eight candidate genes via sequence annotation, haplotype analysis, and tissue expression profiling. Multi-omics approaches have further uncovered the complex genetic architecture underlying rice agronomic and metabolic traits. This strategy provides effective loci for dissecting molecular mechanisms and supports future breeding of ideal-type rice varieties.

## Materials and methods

### Materials

The materials used in this study consisted of 524 and 3,000 diverse rice accessions (**Supplementary Tables 1**, **2**) (Chen et al., 2014; Xie et al., 2015; Dong et al., 2018; Wang et al., 2018b).

### Collection and organization of data

The data pertaining to 12 agronomic traits and 840 metabolic traits of 524 rice varieties, as well as 17 agronomic traits of 3,000 rice varieties, are presented in **Supplementary Tables 3**–**5**, respectively (Chen et al., 2014; Xie et al., 2015; Dong et al., 2018; Wang et al., 2018b). We predicted 11 agronomic traits using predictive models based on 840 metabolic traits across 524 rice accessions (**Supplementary Table 6**). The abbreviations of the agronomic traits mentioned in the text can be found in **Supplementary Table 3**.

### Statistical analysis and correlation analysis

Statistically significant differences between datasets were evaluated by two-way ANOVA. Different letters indicate significant differences at *P* < 0.05. Statistical significance of Pearson’s correlations was shown by ns, no significance; *, *P* < 0.05; **, *P* < 0.01 and ***, *P* < 0.001 (Zhang et al., 2021a, 2022).

### Agronomic-metabolic prediction model

Random Forest (RF), LightGBM, Stepwise regression (SWR), LASSO and Classification and Regression Tree (CART) were employed to construct predictive models for 11 agronomic traits using 840 metabolites.

Random Forest (RF) Model: in this experiment, the basic classification model was built using the default parameters of the randomForest package in R language. Considering the high-dimensional feature data characteristics of rice metabolites, the core hyperparameters were optimized through grid search, with the tuning range set as: n_estimators (200, 300, 500, 800, 1000), mtry (10, 20, 29, 40, 50), nodesize (1, 3, 5), and the optimal parameter was selected based on the maximum R² value of 10-fold cross-validation, eliminating the parameter combinations that are overfitting or underfitting. The rfcv function was called to perform 10-fold cross-validation. Based on the decay trend of cross-validation R², invalid and redundant metabolite features were gradually eliminated, and the core features that contribute significantly to the classification of rice agronomic traits were retained. The significance threshold for feature selection was uniformly set at 0.01 (Breiman, 2001).

LightGBM model: in this experiment, the official default classification parameters of LightGBM were adopted. The model was built based on the gradient boosting decision tree (GBDT) framework. The core default configuration is as follows: 1. The basic iterative model type is boosting_type = gbdt, which adopts the traditional gradient boosting iterative strategy and is suitable for high-dimensional metabolite feature classification tasks; 2. The learning rate is learning_rate = 0.1, and the default number of iterations is n_estimators=100; 3. Tree structure parameters: the maximum tree depth is max_depth = -1 (no depth limit), the minimum number of samples for a single leaf node is min_child_samples = 20, the minimum gain for node splitting is min_split_gain = 0, and the minimum weight sum is min_child_weight = 1e-3; 4. Sampling and regularization parameters: the feature sampling rate is colsample_bytree=1.0, the sample sampling rate is subsample=1.0, the bagging iteration is disabled by default, L1/L2 regularization is disabled, and the initial model has no artificial bias constraints; 5. The early stopping mechanism for iterations is set to be off by default, and the preset number of iterations is fully executed. 6. Considering the high-dimensional and small-sample data characteristics of rice germplasm resources, the parameter tuning range was adjusted through grid search: learning_rate (0.01, 0.05, 0.1, 0.2), n_estimators (100, 200, 300), max_depth (3, 5, 7, -1), min_child_samples (10, 20, 30), colsample_bytree (0.7, 0.8, 0.9, 1.0), aiming to select the parameter combination with the best 10-fold cross-validation R². This avoids the problem of overfitting of high-dimensional features. 7. Based on the 10-fold cross-validation results of rfcv, according to the importance of model features, significant metabolite features with P > 0.01 were excluded, and the high-contribution feature subset was retained. At the same time, the optimal parameter combination was matched to complete the model reconstruction (Ke et al., 2017).

Stepwise regression (SWR) model: in this experiment, the bidirectional stepwise regression algorithm (forward introduction + backward elimination) was adopted. The model was built based on the R language StepReg package, and the experimental significance standards were uniformly adapted. The stability of the stepwise regression model was verified through 10-fold cross-validation. The variable selection results were optimized based on the cross-validation R², and the final feature combination with significant standards met, the best cross-validation fitting effect, was retained to achieve precise dimensionality reduction of high-dimensional metabolite features (Draper, 1998).

LASSO Model: LASSO is an L1 regularized linear regression model, which has the dual functions of feature selection and model fitting. It is modeled based on the default parameters of the glmnet package in R language: 1. The regularization type alpha = 1, pure L1 regularization constraint, which can achieve automatic sparse screening of high-dimensional features; 2. Automatically generates 100 sets of gradient lambda regularization coefficients, covering the strong and weak regularization intervals; 3. Convergence accuracy tol = 1e-7, maximum number of iterations maxit = 1000; 4. Significance screening threshold 0.01, metabolite features with non-zero coefficients and P < 0.01 are considered as effective modeling features; 5. By default, standard normalization processing is enabled, automatically unifying the dimensionalities of 840 metabolite indicators to eliminate interference from numerical magnitude differences. 6. Grid search optimizes the core regularization parameters, tuning range: lambda (0.001, 0.01, 0.1, 1, 5, 10), alpha (0.8, 0.9, 1.0), through 10-fold cross-validation R² to select the optimal parameters, precisely eliminating high-dimensional redundant and noisy metabolite features, and retaining the core predictive variables. 7. Combined with the 10-fold cross-validation results of rfcv, based on the sparse characteristic of LASSO coefficients, features with coefficient shrinkage to 0 and significance P ≥ 0.01 are excluded, constructing a minimalist and efficient subset of rice agronomic trait prediction features (Tibshirani, 1996).

Classification and Regression Tree (CART) Model: this experiment builds a CART model based on the rpart package in R to conduct the classification prediction task for agronomic traits. It fully uses the default parameters provided by the software for initialization of the model, adapting to an 840-dimensional high-dimensional feature set of rice metabolites and a 111-item agronomic trait dataset. Model significance constraint uniformly set as α = 0.01, only allowing metabolite features with significant contributions (P < 0.01) to node splitting, eliminating noise and redundant feature interference; 8. The initial model has no artificial regularization constraints or feature sampling restrictions, fully inputting all metabolite features to ensure no subjective bias in the initial modeling. Combining the characteristics of high-dimensional small sample data of rice germplasm resources, the global grid search optimization of the core hyperparameters of CART is carried out with the optimization goal of maximizing the R² value of tenfold cross-validation (Therneau and Atkinson, 1997).

### Genome-wide association analysis

We used 524 and 3,000 diverse rice accessions to establish two resequencing-based association panels. GWAS was performed for predicted agronomic values using ~6.4 million SNPs (MAF > 0.05) with EMMAX mixed linear models (**Supplementary Table 11**). Significance thresholds were defined as 1, 0.05, and 0.01 divided by the number of SNPs, corresponding to suggestive, significant, and highly significant levels. In this study, a uniform threshold of *P* < 1.0e-05 was applied for both panels, consistent with our laboratory’s previous GWAS on the same 524 accessions and widely accepted for this population (Zhang et al., 2024). SNPs with significant associations within a genomic region were clustered into QTLs, with the SNP showing the lowest P-value defined as the lead SNP. We further identified candidate genes associated with the observed values of 11 agronomic traits and 840 metabolic traits across the 524 rice accessions (Chen et al., 2014; Xie et al., 2015; Dong et al., 2018; Zhang et al., 2021b) and for 17 agronomic traits in the 3,000 accessions (Wang et al., 2018b) obtained from previous studies (**Supplementary Tables 7**, **9**).

### Identification of potential candidate genes

Genes within a 250 kb window flanking each lead SNP were selected as candidate genes (**Supplementary Table 14**). Among these candidates, genes showing differential expression across tissues were identified using the RiceVarMap database (http://ricevarmap.ncpgr.cn/v3/vars_genotype/) (**Supplementary Table 15**) (Xie et al., 2015).

### Gene variation, assessment of variation effect and haplotype analysis

After assessing the effects of SNPs and indels on candidate genes (http://ricevarmap.ncpgr.cn/vars_in_gene/), we searched for functional genome variants using high-impact SNPs and indels (http://ricevarmap.ncpgr.cn/vars) (Xie et al., 2015).

### Different tissues and stress treatment for RT-PCR of candidate genes in Zhonghua 11

The japonica rice cultivar *Zhonghua 11* was used in this study. Seedlings were grown in a Hengli RG-250 growth chamber from sowing to the one-and-a-half-leaf stage under a 14 h light/10 h dark photoperiod, with a photosynthetic photon flux density of 300 μmol m^-^² s^-^¹ and 65%–75% relative humidity. The cultivation soil used for seedling growth was a mixed substrate composed of field dry soil and organic fertilizer: every 180 kg of soil mixture contained 170 kg of dry field soil collected from the experimental field of Yazhou Bay Seed Laboratory and 10 kg of organic fertilizer provided by Jilin Zhongxinyao Agricultural Technology Co., Ltd. On April 10th, 2022, seedlings were individually transplanted into plastic pots (60 cm length × 40 cm width × 25 cm height) filled with 15 kg of the above mixed soil per pot (Guo et al., 2018). The planting density was arranged as 3 individual plants per row and 5 individual plants per column in each pot. After sowing and transplanting, all rice plants were irrigated with conventional water management throughout the growth period. In addition, 500 mL of liquid fertilizer was applied to each pot every two weeks to support the normal growth and development of rice seedlings. The liquid fertilizer was prepared by fully dissolving 370.68 g of ammonium bicarbonate produced by Lanzhou New Area Limin Urea Chemical Co., Ltd., 330.76 g of potassium dihydrogen phosphate produced by Henan Xinzhibo Chemical Products Co., Ltd., and 94.24 g of potassium chloride produced by Jinan Jiushang New Material Technology Co., Ltd. in 60 kg of purified water. For tissue-wide transcriptome profiling, *Zhonghua 11* seedlings at the one-and-a-half-leaf stage were transplanted to an open field in Sanya, Hainan, China. Leaf and stem tissues were harvested at the three-to-four-leaf stage, while flag leaves, inflorescences and panicles were collected at the heading stage. Three-to-four-leaf-stage seedlings were used for all abiotic stress treatments. Drought and salt stresses were imposed via hydroponic culture in Hoagland solution supplemented with 15% PEG-6000 and 150 mM NaCl, respectively, for 24 h. For heat stress, soil-grown seedlings were incubated in the Hengli RG-250 growth chamber at 40 °C (day)/30 °C (night) for 24 h. Cold stress was applied to soil-cultured seedlings at 4 °C (day)/25 °C (night) for 24 h. Untreated control seedlings were maintained at 28 °C (day)/22 °C (night). Notably, drought and salt treatments were performed using a hydroponic system, whereas heat and cold stress treatments adopted soil culture.

Total RNA was extracted from various tissues at different developmental stages by mechanical grinding, using the TaKaRa MiniBEST Universal RNA Extraction Kit (TaKaRa, 9769) according to the manufacturer’s instructions. First-strand cDNA was synthesized from the purified RNA using the PrimeScript 1st Strand cDNA Synthesis Kit (TaKaRa, RR036A), following the protocol provided with the PrimeScript RT Master Mix. The cDNA samples were diluted 10-fold, and quantitative real-time PCR (qRT-PCR) was performed to amplify target genes and the actin reference gene. Amplification was conducted using the SYBR Premix Taq II Kit (TaKaRa, RR036A) according to the manufacturer’s protocol. The thermal program was: 95 °C for 5 min; 40 cycles of 95 °C for 15 s, 57 °C for 15 s, 62 °C for 30 s. Amplicon purity was confirmed by a single distinct peak in melting curve analysis. qRT-PCR primers are listed **Supplementary Table 16** and designed using the online OligoAnalyzer™ by Integrated DNA Technologies (https://sg.idtdna.com/pages/tools/), and qRT-PCR data were analyzed via the method proportional to *Actin* (Gene ID: Os03g0718100).

## Results

### Colocalization resolution for manual measurement value among agronomic traits and metabolites in two rice association populations

Comparing GWAS results across traits in 524 diverse accessions, we detected co-localization of multiple loci between agronomic traits and metabolites (**Supplementary Figures 1**, **2**; **Supplementary Tables 7**, **8**). For instance, Grain thickness_a and Grain width_a shared two co-localized SNPs (sf0901890440, sf0902135055); Grain weight_a and Grain_length_a shared two SNPs (sf0202346296, sf0316705162); and Grain thickness_a and Grain weight_a shared one SNP (sf0827601454). In the 3,000 rice accessions, co-localization was also observed (**Supplementary Figure 1**; **Supplementary Tables 9**, **10**): 343 co-localized SNPs among Grain length_b, width_b, weight_b, and seven between Leaf length_b and width_b. Cross-population comparisons revealed further co-localization (**Supplementary Figure 1**; **Supplementary Table 10**): three SNPs between Grain length and width across populations, one SNP (sf0316705162) between Grain weight_a and weight_b, two SNPs between Grain thickness and width, and multiple shared SNPs across grain traits (sf0505373357, sf0316705162, sf0505372933). Co-localization also occurred between metabolites and agronomic traits (**Supplementary Figure 2**; **Supplementary Table 8**): one SNP (sf0113095158) linked spikelet_length_a with two flavonoids; one (sf0233300979) between yield_a and metabolite mr1977; one (sf0303533203) between yield_a and sinapoyl quinic acid. By integrating tissue-specific expression, we identified seven major-effect co-localized QTLs containing 45 candidate genes for agronomic and metabolic traits (**Supplementary Table 10**). These co-localizations clarified genotype–phenotype relationships in two rice panels. Reliable co-localized SNPs and genes provide valuable resources for breeding and dissecting the genetic and molecular architecture of agronomic and metabolic traits.

### Metabolites as biomarkers to predict traditional agronomic traits

**Figure 1:**
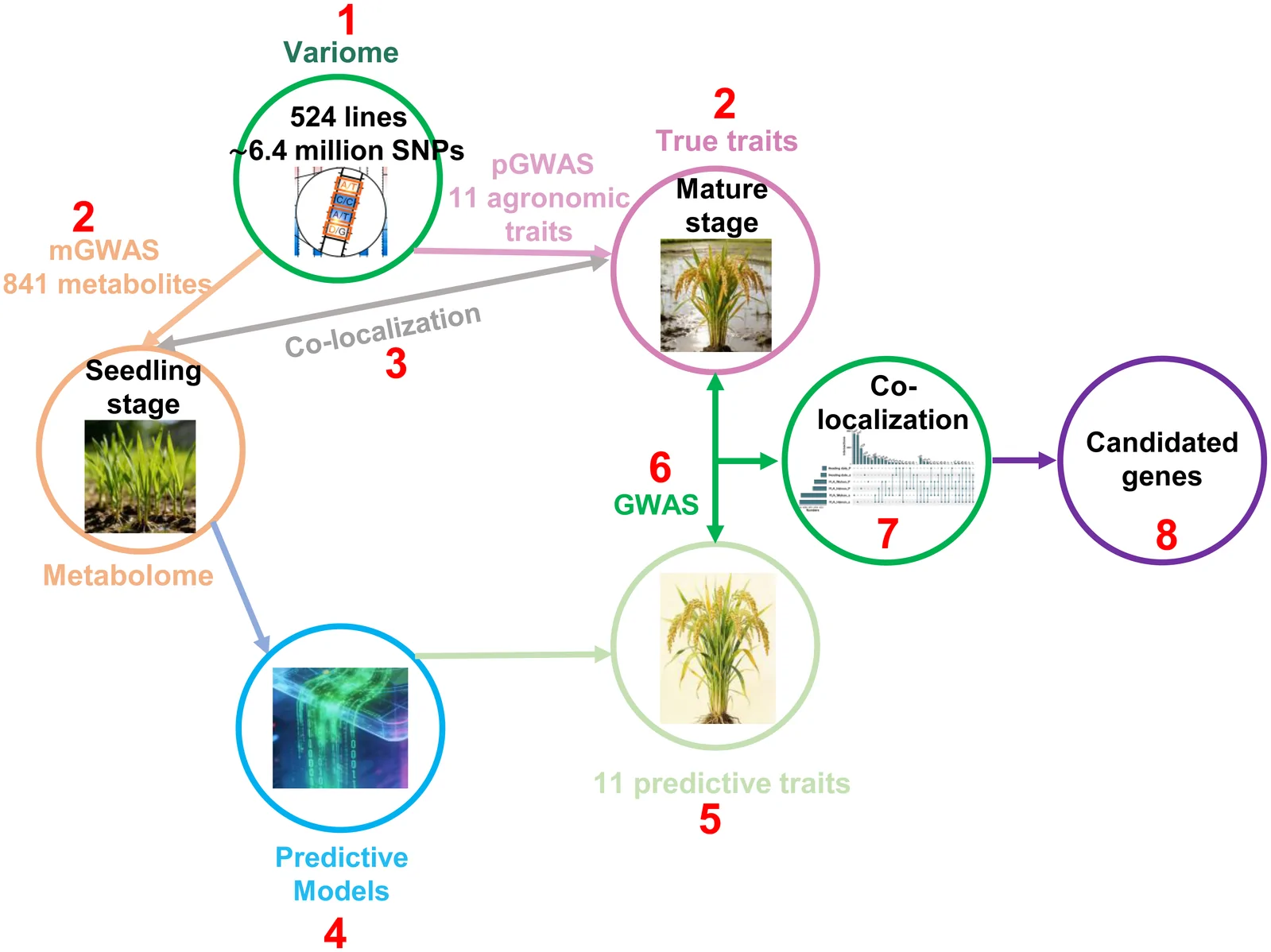
Technical route of this study. The sequence follows step 1 to 8. Step 1. Variome: genotyping of 524 rice accessions generated ~6.4 million SNPs. Step 2. mGWAS (GWAS for 841 metabolites) and pGWAS (Eleven agronomic traits). Step 3. Co-location analysis: co-location analysis to mGWAS and pGWAS. Step 4. Predictive models: construction of predictive models relying on seedling metabolite data. Step 5. Predictive traits: eleven agronomic traits predicted by the seedling metabolome models. Step 6. pGWAS: co-location analysis for phenotype-based GWAS for both measured true traits and predicted traits. Step 7. Co-localization: genetic co-localization analysis of loci for metabolites, true traits and predicted traits. Step 8. Candidate genes: identification of candidate genes residing within co-localized loci.

To dissect the complex relationships between agronomic traits and metabolites, we comprehensively employed stepwise regression (SWR), random forest (RF), LASSO, CART and LightGBM models to establish agronomic trait-metabolite prediction models based on the observed values of agronomic traits and metabolite data, ultimately generating predicted values for 11 agronomic traits. Subsequently, genome-wide association studies (GWAS) were conducted separately on the observed values, predicted values of agronomic traits, and 841 metabolites. Multiple hotspot regions co-localized with agronomic and metabolite traits were identified, from which candidate genes were screened (**Figure 1**).

**Figure 2:**
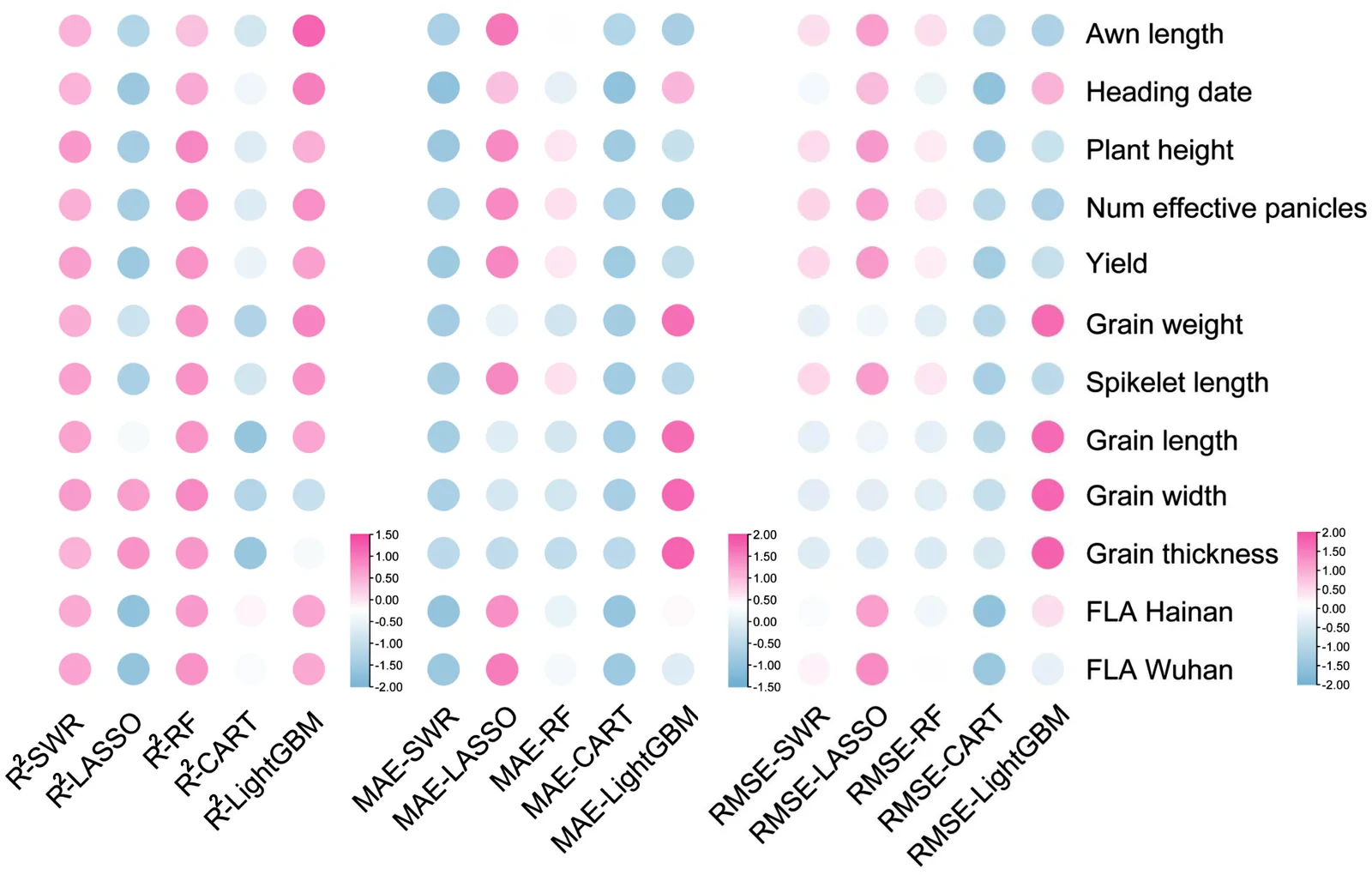
Comparison of relevant parameters for multi-model prediction. Comparison of model predictive performance across rice agronomic traits visualized by dot heatmap. To enable cross-model comparison among metrics with different scales, all raw R^2^, MAE and RMSE values were subjected to row-wise Z-score standardization within each trait row. X-axis: evaluation metrics paired with five predictive algorithms. Y-axis: rice agronomic traits including yield, grain traits, plant architecture and flowering-related traits. SWE, Stepwise regression model; LASSO, LASSO model; RF, Random forest model; CART, CART model; LightGBM, LightGBM model; R^2^, Coefficient of determination; RMSE, Root Mean Square Error; MAE, Mean absolute error.

We developed a series of predictive models for traditional agronomic traits, including plant height, yield, flag leaf angle (FLA), grain length, thickness, weight, width, heading date, number of effective panicles, and spikelet length, using predictive models. The coefficient of determination (R^2^) was adopted as the primary metric to quantify model predictive performance, where a higher R^2^ value denotes superior model fitting capacity, while negative R^2^ values reflect low predictive reliability. For awn length, heading date, grain weight and spikelet length, the LightGBM algorithm yielded the optimal predictive performance, with corresponding R^2^ values of 0.4749, 0.4608, 0.1950 and 0.1593, which outperformed all alternative models. For plant height, the SWR model achieved the highest R^2^ of 0.3654. RF model exhibited the best fitting performance for the remaining traits including the number of effective panicles, grain yield, grain length, grain width, FLA Hainan and FLA Wuhan, with R^2^ values of 0.2810, 0.2637, 0.1623, 0.4205, 0.1203 and 0.1519, respectively. In terms of grain thickness, the LASSO regression model provided the optimal fit, with an R^2^ of 0.2433. Collectively, most agronomic traits produced negative R^2^ values when modeled via LASSO and CART, revealing their poor generalization capacity. By contrast, LightGBM, RF and SWR exhibited trait-specific predictive advantages and can be prioritized according to target agronomic characteristics (**Figure 2**; **Supplementary Figures 3**–**5**; **Supplementary Table 6**). Thus, metabolites at the terminal seedling stage are insufficient for accurately predicting yield using a linear mixed model.

In contrast, these predictive models performed well in forecasting other agronomic traits, with strong correlations between predicted and observed values. Seedling-stage metabolites therefore represent a key resource for predicting agronomic traits across multiple growth stages and can serve as biomarkers for traditional agronomic traits, supporting the potential use of predictive models in predicting rice agronomic characteristics.

### GWAS analysis of predictive models predicted agronomic trait values

**Figure 3:**
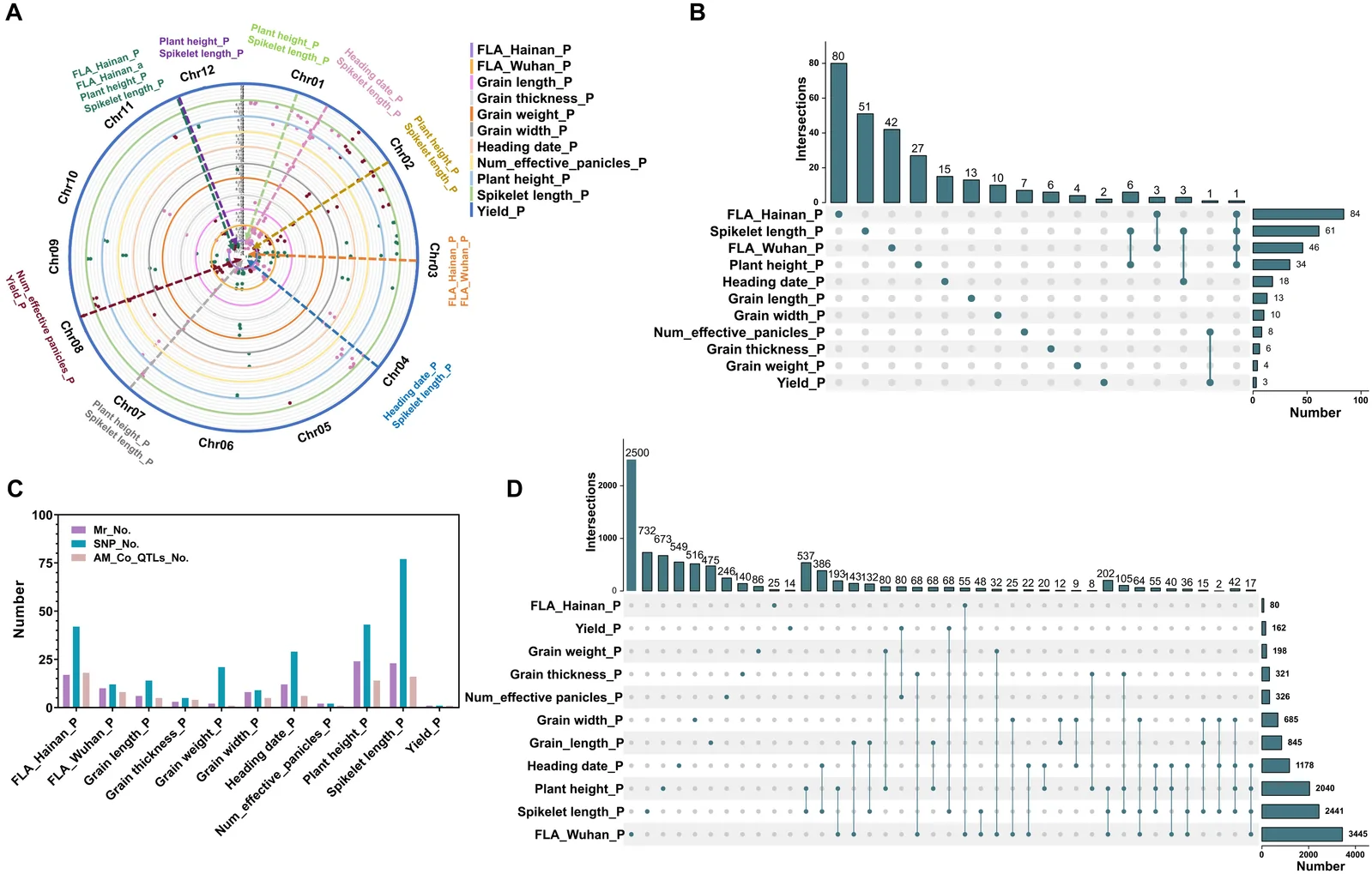
Candidate genes mining for predictive value of agronomic traits. **(A)** Manhattan plots showed nine hotspots by GWAS for the predictive value of agronomic traits among plant height, heading stage, FLA_Hainan, FLA_Wuhan, spikelet length, grain width, grain length, grain thickness, 1000-grain weight, number of effective panicles and yield. These points represent log-transformed P-values from GWAS (P-values ≤ 1.0e-05). Descriptions of all agronomic traits and their abbreviations can be found in **Supplementary Table 3**. **(B)** The VENN figure shows the co-located SNPs by GWAS among plant height, heading stage, FLA_Hainan, FLA_Wuhan, spikelet length, grain width, grain length, grain thickness, 1000-grain weight, number of effective panicles and yield. **(C)** The bar chart shows the co-located numbers of SNPs, metabolites, and co-located the numbers of QTLs for the predictive value of agronomic traits among plant height, heading stage, FLA_Hainan, FLA_Wuhan, spikelet length, grain width, grain length, grain thickness, 1000-grain weight, number of effective panicles and, yield, respectively. **(D)** The VENN figure showed the co-located genes within 250 kb before and after the lead SNPs among plant height, heading stage, FLA_Hainan, FLA_Wuhan, spikelet length, grain width, grain length, grain thickness, 1000-grain weight, number of effective panicles and yield.

By comparing the GWAS results of predictive models for predicted agronomic traits values with those of 524 diverse accessions and integrating GWAS results for the true values of all agronomic traits from two rice association populations, we identified 57 hotspots (**Supplementary Figure 6**; **Supplementary Tables 11**, **12**). This confirms that the predictive models-based method is effective in predicting true values to obtain reliable QTLs and candidate genes associated with complex agronomic traits. Additionally, GWAS of predicted agronomic trait values revealed seven credible co-localized hotspots (**Figure 3A**; **Supplementary Table 12**). For instance, two co-localized SNPs (12_3637197, 12_3638037) within QTL *qP.C12.3* were identified among Plant height_P, Spikelet length_P, and FLA_Hainan_P; six co-localized SNPs (1_19388316, 12_3637197, 12_3637715, 2_16438680, 2_16724980, 7_19793419) within QTLs *qP.C01.2*, *qP.C02.4*, *qP.C07.1*, and *qP.C12.3* were found between Plant height_P and Spikelet length_P. Furthermore, FLA_Hainan_P and FLA_Wuhan_P shared one co-localized SNP (3_15075278) within QTL *qP.C03.3*, whereas three co-localized SNPs (4_21176154, 1_27543151, 4_21448025) within QTLs *qP.C01.3* and *qP.C04.2* were identified between Heading_date_P and Spikelet_length_P. Only one co-localized SNP (8_20170844) within QTL *qP.C08.1* was detected between Num_effective panicles_P and Yield_P (**Figure 3B**; **Supplementary Table 12**).

Notably, the number of co-localized SNPs and metabolites used by predictive models to predict agronomic traits varied (**Figure 3C**). Specifically, predicted values for plant height, spikelet length, leaf angle, and heading date remained consistently high across variations in metabolite indicators, significant SNPs, agronomic indicators, and co-localized metabolite candidate SNPs. In contrast, the yield-related predicted values decreased significantly under all variations. In conclusion, colocalization analysis of candidate genes, encompassing a 250kb region upstream and downstream of identified hotspots, identified 31 candidate genes significantly affected by differential expression across tissues (**Figure 3D**; **Supplementary Tables 13**, **14**). These credible co-localized loci and genes provide valuable references for breeders and lay a foundation for understanding molecular mechanisms and breeding ideal plant-type rice varieties.

### Identification of co-localized candidate genes regulating the plant height, spikelet length, grain length and FLA Traits on Chr01 and Chr02

**Figure 4:**
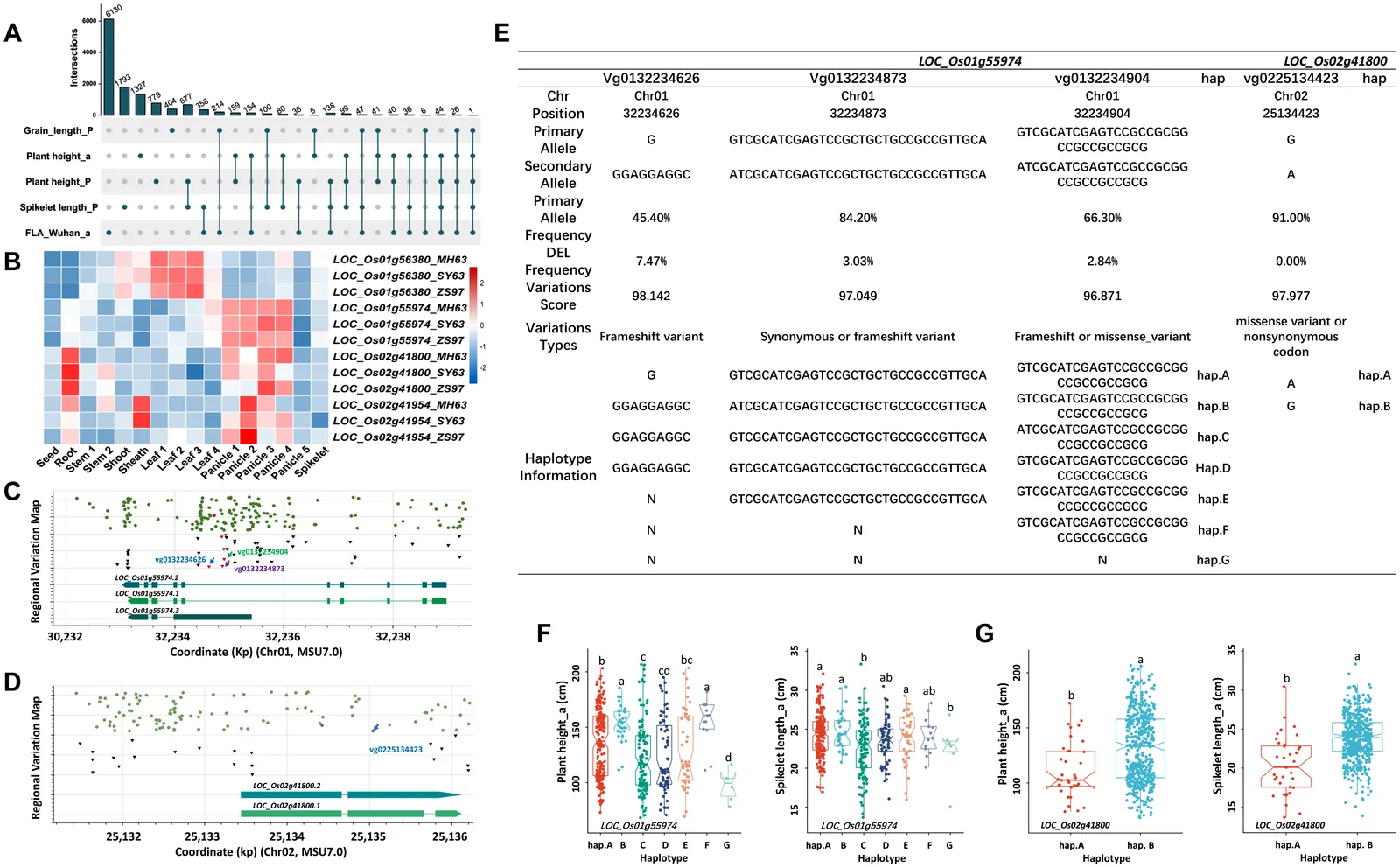
Analysis of candidate genes by GWAS for colocalization information associated with the predictive and true value among plant height, spikelet length, grain length and FLA. **(A)** The VENN figure shows the co-located genes within 250 kb of upstream and downstream the lead SNPs associated with the predictive and true value among plant height, spikelet length, grain length and FLA. **(B)** Expression levels of the co-located candidate genes associated with plant height, spikelet length, grain length or FLA in different tissues and periods of the rice cultivars ‘*MH63*’, ‘*SY63*’ and ‘*ZS97*’. The expression levels of candidate genes in different tissues were row-wise Z-score standardized. **(C)** Variation Plots of *LOC_Os01g55974*. Dots represent SNP variants, but triangles represent indel variants. Red dot and triangles represent high impact variants. Green dots and black triangles represent low impact variants. **(D)** Variation plots of *LOC_Os02g41800*. Dots represent SNP variants, but triangles represent indel variants. Purple dots represent high impact variants. Green dots and black triangles represent low impact variants. **(E)** Detailed variation information including of significant indels variation and haplotypes information of *LOC_Os01g55974* and *LOC_Os02g41800*. **(F, G)** Boxplots of haplotypes of *LOC_Os01g55974* **(F)** and *LOC_Os02g41800* **(G)** of the plant height and spikelet length, respectively. These comparisons were conducted at the individual gene level. Different letters (a, ab, b, bc, c, cd and d) indicate significant difference at P < 0.05 using two-way ANOVA.

Identifying co-localized candidate genes for complex traits remains a challenge in genetic studies. We focused on key hotspots on chromosomes Chr01 and Chr02 underlying four important rice traits: plant height, spikelet length, grain length, and FLA_Wuhan (**Figure 4A**; **Supplementary Tables 13**, **14**). Using sequence variation annotation, haplotype analysis, and tissue-specific differential expression profiling, we identified four candidate genes, several of which exhibited strong tissue-specific expression (**Supplementary Table 15**). We highlighted *LOC_Os01g55974* and *LOC_Os02g41800*, whose functions in rice have not been previously reported.

*LOC_Os01g55974* is located in a key hotspot on chromosome Chr01 and shows strong associations with multiple phenotypic traits, including Plant height_P, Grain length_P, Spikelet length_P, FLA_Wuhan_a, and Plant height_a, which together contribute to plant growth regulation (**Figure 4A**; **Supplementary Table 14**). This gene encodes a dCMP deaminase that belongs to the cytidine deaminase family, designated *Oryza sativa STRIPE2* (*OsSTRIPE2*). It catalyzes the deamination of dCMP to dUMP and is essential for chloroplast development and chlorophyll biosynthesis in rice. *OsSTRIPE2* mutants display leaf streaking, reduced chlorophyll content, and abnormal chloroplast structures, which can be rescued by exogenous dUMP (Xu et al., 2014). Analysis of RNA-seq data from ZS97, SY63, and MH63 across tissues and developmental stages revealed that *OsSTRIPE2* is highly expressed in panicles at multiple stages, supporting its role in grain-related traits (**Figure 4B**; **Supplementary Table 15**). *OsSTRIPE2* contains 10 exons and 9 introns, with high-impact SNP/InDel variants (vg0132234626, vg0132234873, vg0132234904) causing frameshift, missense, and nonsynonymous mutations (**Figures 4C**, **E**). Haplotype analysis identified seven haplotypes (hap.A-G). Plant height and spikelet length differed significantly among haplotypes: hap.B accessions had significantly taller plants and longer spikelets, whereas hap.G accessions were shorter with smaller spikelets (**Figure 4F**). Thus, we classified hap.B as a tall-long spikelet haplotype and hap.G as a short-small spikelet haplotype. These results indicate that *OsSTRIPE2* plays a pivotal role in regulating rice plant height and spikelet length.

*LOC_Os02g41800*, situated on the Chr02 hotspot, overlaped with Grain length_P, Spikelet length_P, Spikelet length_a, and FLA_Hainan_P (**Figure 4A**; **Supplementary Table 14**). *LOC_Os02g41800*, designated as *Oryza sativa AUXIN RESPONSE FACTOR 8* (*OsARF8*), encodes an auxin response factor and is homologous to *Arabidopsis thaliana AUXIN RESPONSE FACTOR 16* (*AtARF16*, *AT4G30080*). Although *OsARF8* may function in root cap cell differentiation, its precise biological roles remain uncharacterized. RNA-seq analysis across tissues and developmental stages in ZS97, SY63, and MH63 revealed that *OsARF8* is highly expressed in roots and panicles, strongly suggesting a role in root and grain-related traits (**Figure 4B**; **Supplementary Table 15**). *OsARF8* contains two exons and one intron, with a high-impact variant (vg0225134423) causing missense/nonsynonymous mutations (**Figures 4D, E**). Haplotype analysis identified two haplotypes: hap.A and hap.B. Hap.B accessions exhibited significantly taller plants and longer spikelets than hap.A (**Figure 4G**). Thus, we classified hap.B as a tall-long spikelet haplotype and hap.A as a short-small spikelet haplotype. These results indicate that *OsARF8* plays a pivotal role in regulating rice plant height and spikelet length.

This study provides deeper insights into the genetic mechanisms governing these traits, laying the foundation for more precise, targeted breeding strategies to develop elite rice varieties with optimized plant height, spikelet length, grain length, and FLA. In conclusion, our identification of co-localized candidate genes on chromosomes Chr01 and Chr02 advances understanding of the genetic regulation of plant height, spikelet length, grain length, and FLA. This represents a valuable frontier in plant genetics.

### Identification of co-localized candidate genes for heading stage and FLA in the hotspot regions on Chr06 and Chr08

**Figure 5:**
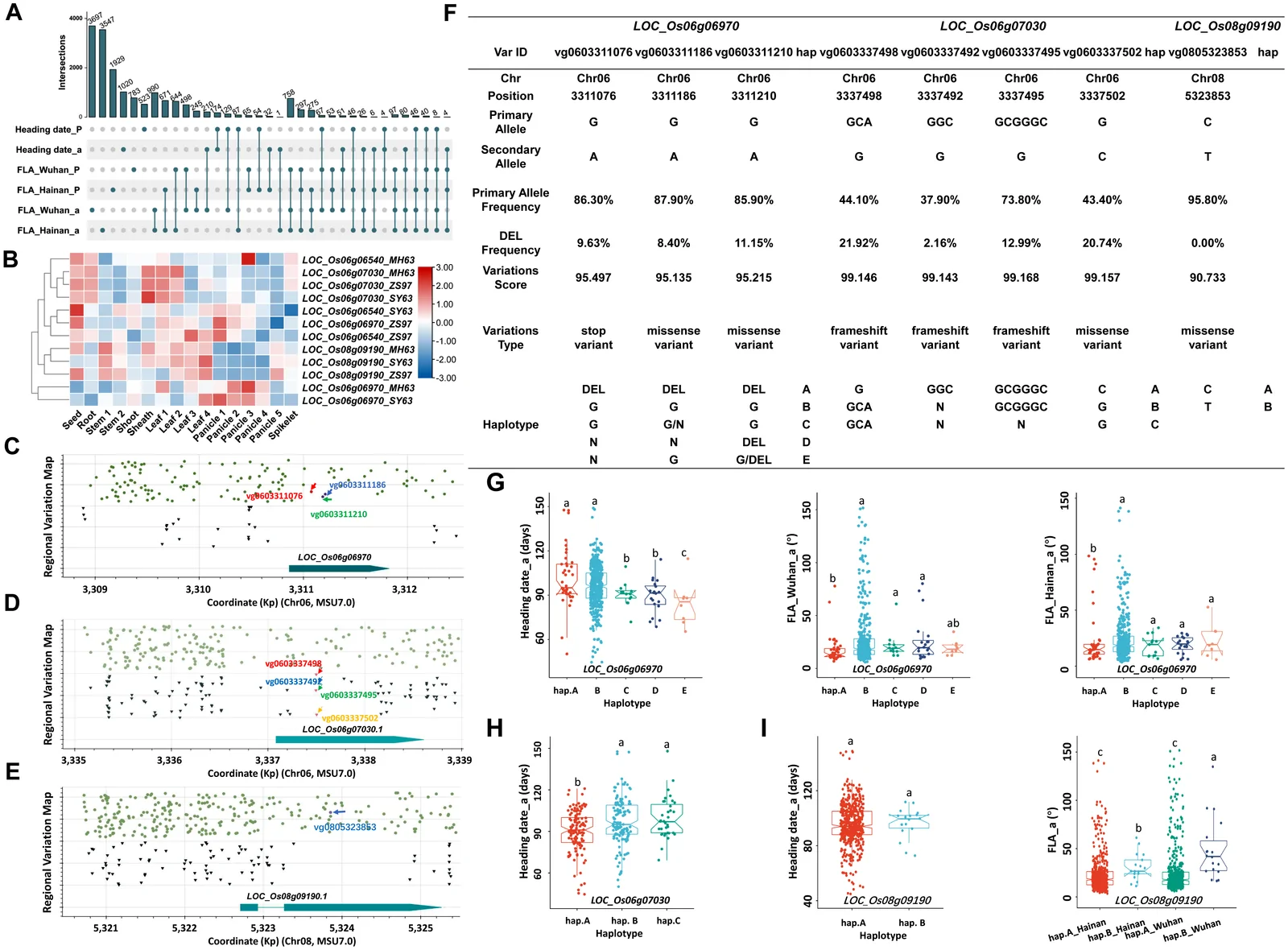
Analysis of candidate genes by GWAS for colocalization information associated with the predictive and true value of heading date and FLA. **(A)** The VENN figure shows the co-located genes within 250 kb of upstream and downstream the lead SNPs associated with the predictive and true value between heading date, FLA_Hainan and FLA_Wuhan. **(B)** Expression levels of the co-located candidate genes associated with the predictive and true value of heading date and FLA in different tissues and periods of the rice cultivars ‘*MH63*’, ‘*SY63*’ and ‘*ZS97*’. The expression levels of candidate genes in different tissues were row-wise Z-score standardized. **(C)** Variation plots of *LOC_Os06g06970*. Dots represent SNP variants, but triangles represent indel variants. Red and purple dots represent high impact variants. Green dots or black triangles represent low impact variants. **(D)** Variation plots of *LOC_Os06g07030*. Dots represent SNP variants, but triangles represent indel variants. Red triangles represent high impact variants. Green dots or black triangles represent low impact variants. **(E)** Variation plots of *LOC_Os08g09190*. Dots represent SNP variants, but triangles represent indel variants. Purple dot represents high impact variants. Green dots or black triangles represent low impact variants defined by SNPs or indels, respectively. **(F)** Detailed variation information and haplotypes information including of significant indels variation and haplotypes information of *LOC_Os06g06970*, *LOC_Os06g07030* and *LOC_Os08g09190.* **(G–I)**. Boxplots of haplotypes of *LOC_Os06g06970* **(G)**, *LOC_Os06g07030* **(H)** and *LOC_Os08g09190* **(I)** of the heading date and FLA, respectively. These comparisons were conducted at the individual gene level. Different letters (a, ab, b and c) indicate significant difference at P < 0.05 using two-way ANOVA.

Based on previous results, we focused on two hotspots on Chr06 and Chr08 associated with heading date, FLA_Hainan, and FLA_Wuhan (**Figure 5A**; **Supplementary Tables 13**, **14**). Using sequence variation annotation, haplotype analysis, and tissue-specific differential expression profiling, we identified four candidate genes within the 250kb flanking regions of the hotspots. Several genes showed strong tissue-specific expression (**Figure 5B**; **Supplementary Table 15**). We further prioritized *LOC_Os06g06970*, *LOC_Os06g07030* and *LOC_Os08g09190*, the functions of which in rice have not been previously reported.

*LOC_Os06g06970*, designated as *Oryza sativa DEHYDRATION-RESPONSIVE ELEMENT BINDING 1D* (*OsDREB1D*), mapped to the hotspot *qP.C06.2* and co-localized with Heading date_P, Heading date_a, and FLA_Hainan_P. This gene encodes an AP2/EREB transcription factor (**Figure 5A**; **Supplementary Table 14**). Previous studies reported that *OsDREB1D* functions mainly in drought, salt, and cold stress responses, but its other biological roles remain uncharacterized (Dubouzet et al., 2003; Mao et al., 2012). RNA-seq analysis across tissues and developmental stages in ZS97, SY63, and MH63 revealed that *OsDREB1D* was highly expressed in panicles and leaves, suggesting a role in plant architecture and grain-related traits (**Figure 5B**; **Supplementary Table 15**). *OsDREB1D* contains one exon and harbors multiple high-impact SNP/InDel variants (vg0603311076, vg0603311186 and vg0603311210) causing frameshift, stop-gain, nonsynonymous, and missense mutations (**Figures 5C**, **F**). Haplotype analysis identified five haplotypes (hap.A-E). Hap.A and hap.B showed significantly later heading (Heading stage_a), while hap.E exhibited earlier heading than other haplotypes (**Figure 5G**). Hap.A had lower FLA values in both Hainan and Wuhan. Notably, hap.B showed significantly later heading and larger FLA in both environments. Hap.E displayed early heading but large FLA. Thus, we classified hap.B as a late-heading, large-FLA haplotype and hap.E as an early heading, large-FLA haplotype. These results indicate that *OsDREB1D* contributes to heading date and FLA regulation in rice.

*LOC_Os06g07030* mapped to hotspot *qP.C06.2* and co-localized with Heading date_P, Heading date_a, and FLA_Hainan_P (**Figure 5A**; **Supplementary Table 14**). This gene encodes an AP2-domain protein, and its biological functions have not yet been reported. The gene is designated as *Oryza sativa DEHYDRATION-RESPONSIVE ELEMENT BINDING FACTOR 4* (*OsDEAR4*), which is homologous to *Arabidopsis thaliana DEAR4* (*AtDEAR4*, *AT4G36900*) and belongs to the ERF/AP2 transcription factor family, DREB subfamily A-5. RNA-seq analysis across tissues and developmental stages in ZS97, SY63, and MH63 revealed that *OsDEAR4* is highly expressed in seeds, roots, leaves, and sheaths, suggesting its role in plant architecture and grain-related traits (**Figure 5B**; **Supplementary Table 15**). *OsDEAR4* contains a single exon and harbors multiple high-impact SNP/InDel variants (vg0603337492, vg0603337495, vg0603337498, vg0603337502) causing frameshift, missense, and nonsynonymous mutations (**Figure 5D**). Haplotype analysis identified three haplotypes: hap.A, hap.B, and hap.C (**Figure 5F**). Hap.B and hap.C showed significantly later heading (Heading stage_a) than hap.A (**Figure 5H**). Thus, we classified hap.B and hap.C as late heading haplotypes and hap.A as an early heading haplotype. These results indicate that *OsDEAR4* contributes to the regulation of heading date in rice.

*LOC_Os08g09190* encodes an auxin efflux carrier family protein and maps to hotspot *qP.C08.4*, co-localizing with Heading date_P, FLA_Hainan_a, FLA_Wuhan_a, and Heading date_a (**Figure 5A**; **Supplementary Table 14**). However, its biological functions remain unknown. This gene, designated *Oryza sativa PIN-LIKES 2* (*OsPILS2*), shows homology with *Arabidopsis thaliana PIN-LIKES 2* (*AtPILS2*). RNA-seq analysis of tissues and developmental stages in ZS97, SY63, and MH63 revealed that *OsPILS2* was highly expressed in seeds, stems, sheaths, and leaves, suggesting a role in plant architecture and grain-related traits (**Figure 5B**; **Supplementary Table 15**). *OsPILS2* contains two exons and one intron, with a high-impact SNP/InDel variant (vg0805323853) that causes nonsynonymous and missense mutations (**Figure 5E**). Haplotype analysis identified two haplotypes: hap.A and hap.B (**Figure 5F**). Heading stage_a did not differ significantly between hap.A and hap.B. However, hap.B showed significantly larger FLA than hap.A in both Hainan and Wuhan. Notably, FLA values for hap.B were significantly higher in Wuhan than in Hainan (**Figure 5I**). Thus, we classified hap.B as a large-FLA haplotype and hap.A as a small-FLA haplotype, with hap.B affected by environmental conditions. These results indicate that *OsPILS2* contributes to FLA regulation in rice. Therefore, we inferred that *OsPILS2* was most likely involved in the heading stage and FLA in rice.

This study aimed to identify co-localized candidate genes within these hotspots, providing insights into the genetic mechanisms underlying heading date and FLA. We detected multiple candidate genes in the Chr06 and Chr08 hotspots, implying functional coordination.

### Identification of co-localized candidate genes underlying grain width hotspots on Chr09 and Chr10

**Figure 6:**
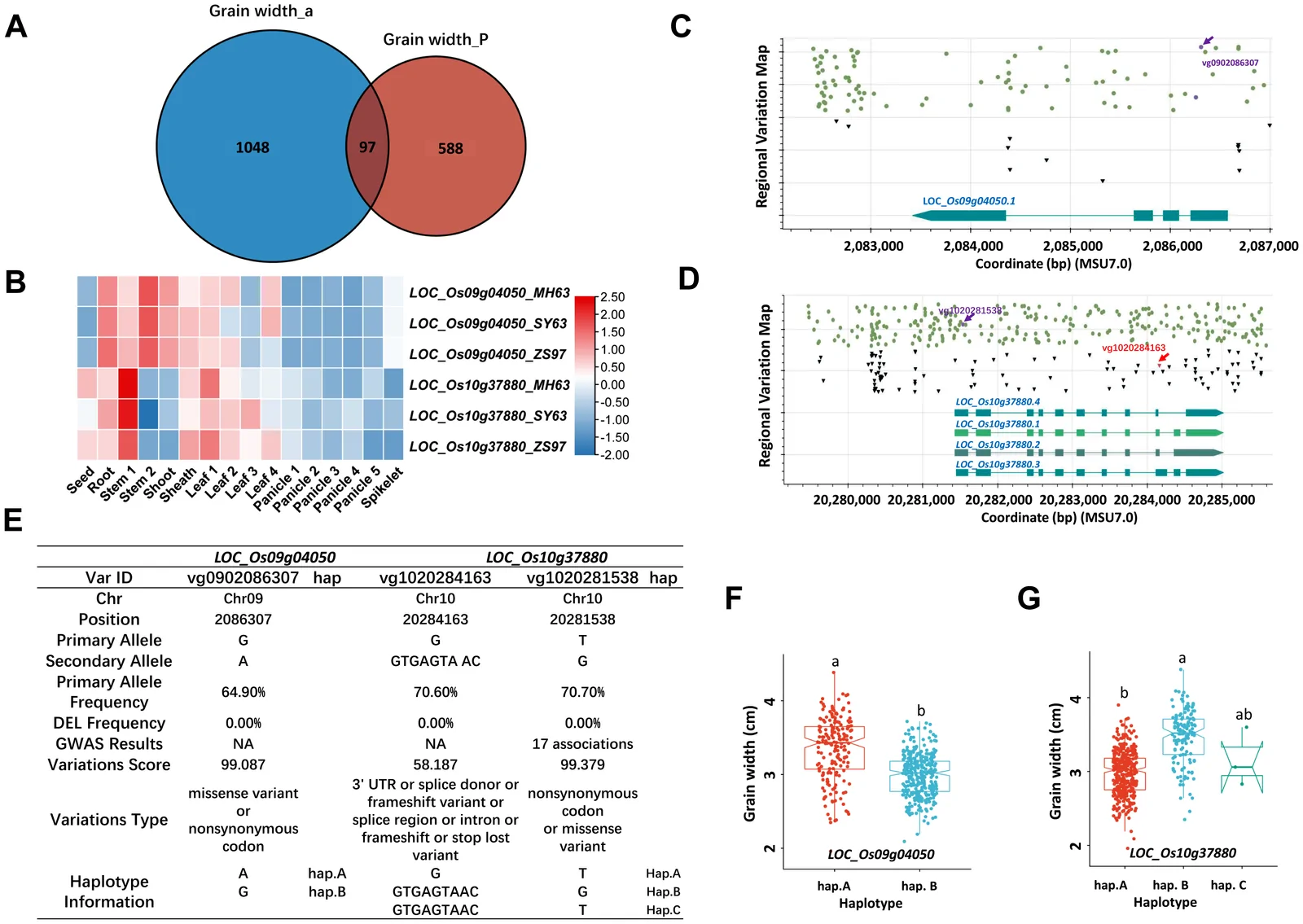
Analysis of candidate genes by GWAS for colocalization information associated with the predictive and true value of the grain width. **(A)** The VENN figure shows the co-located genes within 250 kb of upstream and downstream the lead SNPs associated with the predictive and true value of the grain width. **(B)** Expression levels of the candidate genes associated with the predictive and true value of the grain width. in different tissues and periods of the rice cultivars ‘*MH63*’, ‘*SY63*’ and ‘*ZS97*’. The expression levels of candidate genes in different tissues were row-wise Z-score standardized. **(C)** Variation plots of *LOC_Os09g04050*. Dots represent SNP variants, but triangles represent indel variants. Purple dots represent high impact variants. Green dots and black triangles represent low impact variants. **(D)** Variation plots of *LOC_Os10g37880*. Dots represent SNP variants, but triangles represent indel variants, respectively. Purple dot and red triangle represent high impact variants. Green dots and black triangles represent low impact variants defined by SNPs and indels, respectively. **(E)** Detailed variation information including of significant indels variation and haplotypes information of *LOC_Os09g04050* and *LOC_Os10g37880*. **(F, G)** Boxplots of haplotypes of *LOC_Os09g04050* **(F)** and *LOC_Os10g37880* **(G)** of the grain width. These comparisons were conducted at the individual gene level. Different letters (a, ab and b) indicate significant difference at P < 0.05 using two-way ANOVA.

Based on previous findings, we focused on two critical grain width-associated regions on Chr09 and Chr10 (**Figure 6A**; **Supplementary Tables 13**, **14**). *LOC_Os09g04050* and *LOC_Os10g37880* were located within the 250 kb flanking intervals of the respective hotspots. These genes were identified via sequence variant annotation, haplotype analysis, and tissue-specific expression profiling in ZS97, SY63, and MH63. They may function importantly in the growth and development of multiple rice tissues and organs, although their precise functions remain uncharacterized.

*LOC_Os09g04050* is located within hotspot *qP.C09.3* and overlaps with Grain width_P and Grain width_a, although its biological function remains unknown (**Figure 6A**; **Supplementary Table 14**). *LOC_Os09g04050*, designated as *Oryza sativa CINNAMOYL-CoA REDUCTASE 1* (*OsCCR1*), is homologous to *Arabidopsis thaliana CINNAMOYL-CoA REDUCTASE 1* (*AtCCR1*, *AT1G15950*), which encodes a cinnamoyl-CoA reductase involved in lignin biosynthesis. RNA-seq analysis across tissues and developmental stages in ZS97, SY63 and MH63 revealed that *OsCCR1* was highly expressed in roots, stems, shoots, sheaths and leaves, implying an important role in plant architecture and grain morphology (**Figure 6B**; **Supplementary Table 15**). *OsCCR1* consists of four exons and three introns, and carries a high-impact variant (vg0902086307) leading to a missense nonsynonymous mutation (**Figure 6C**). Haplotype analysis identified two major haplotypes, hap.A and hap.B (**Figure 6E**). Accessions with hap.A displayed significantly wider grains than those with hap.B (**Figure 6F**). Therefore, we defined hap.A as a wide grain haplotype and hap.B as a narrow grain haplotype. These results support that *OsCCR1* contributes to the regulation of grain width in rice.

*LOC_Os10g37880*, is located in the key region *qP.C10.2* and overlaps with Grain width_P and Grain width_a. This gene encodes a protein belonging to the oxygenase superfamily, although its precise biological function remains uncharacterized (**Figure 6A**; **Supplementary Table 14**). *LOC_Os10g37880*, designated as *Oryza sativa 2-oxoglutarate* (*OsOG1*), showed homology to *Arabidopsis thaliana AT4G16765*, implying functional conservation across plant species. RNA-seq analysis of ZS97, SY63 and MH63 across diverse tissues and developmental stages revealed that *OsOG1* was highly expressed in seeds, roots, stems, sheaths and leaves, supporting its important role in plant morphology and grain development (**Figure 6B**; **Supplementary Table 15**). *OsOG1* comprises ten exons and nine introns, and harbors high-impact SNP/InDel variants (vg1020281538, vg1020284163) resulting in frameshift, splice region, stop-loss, missense and nonsynonymous mutations (**Figure 6D**). Haplotype analysis identified three major haplotypes (**Figure 6E**). Accessions carrying hap.B displayed significantly wider grain width_a than those with other haplotypes (**Figure 6G**). Thus, we defined hap.B as a wide-grain haplotype and hap.A as a narrow-grain haplotype. Collectively, these findings demonstrate that *OsOG1* contributes to the regulation of in rice.

Given the complex genetic basis of cereal crops, identifying co-localized candidate genes is essential for dissecting grain width. We targeted hotspot regions on chromosomes 9 and 10 containing grain width loci and predicted candidate genes via a multi-step approach.

### Tissue-specific and stress-responsive expression profiles of seven candidate genes

**Figure 7:**
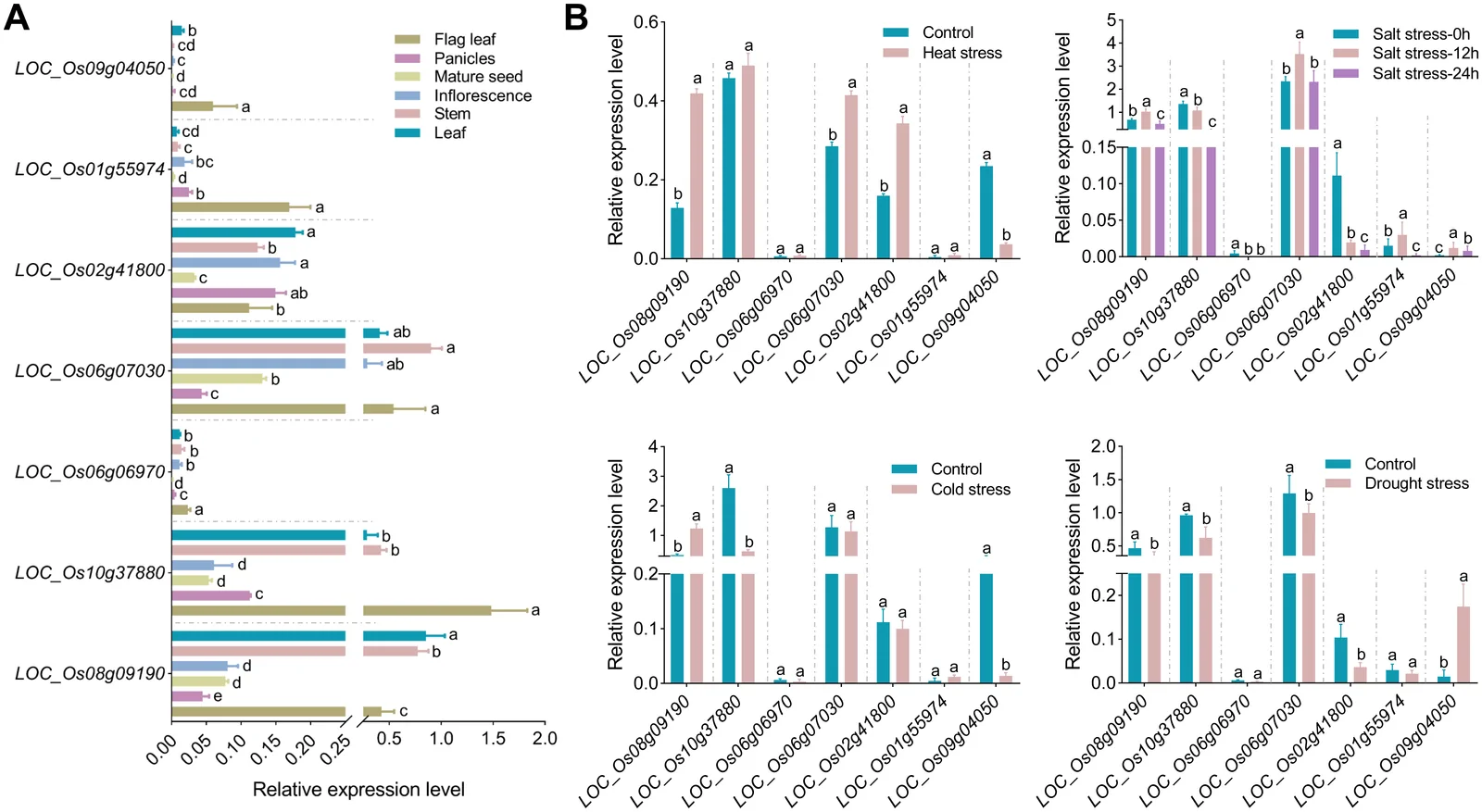
Relative expression profiles by qRT-PCR of seven candidate genes across different tissues and stress conditions. **(A)** The figure depicts the relative expression patterns of seven candidate genes across different tissues, including leaves, stems, flag leaves, panicles, inflorescences, and mature seeds. These comparisons were conducted at the individual gene level. Different letters (a, ab, b, bc, c, cd and d) indicate significant difference at P < 0.05 using two-way ANOVA. **(B)** These figures illustrate the relative expression patterns of seven candidate genes under heat, cold, salt, and drought stress conditions, respectively. These comparisons were conducted at the individual gene level. Different letters (a, b and c) indicate significant difference at P < 0.05 using two-way ANOVA.

To elucidate the spatiotemporal expression patterns of the seven candidate genes identified in this study, we systematically characterized their tissue-specific transcriptional profiles across multiple rice organs using RT-PCR (**Figure 7A**). Among these genes, *LOC_Os02g41800* (*OsARF8*) exhibited significantly higher expression in leaves, inflorescences, and panicles than in other tissues (e.g., roots, stems and sheaths). In contrast, *LOC_Os01g55974* (*OsSTRIPE2*) showed a distinct expression pattern, with preferential accumulation in flag leaves-the primary photosynthetic organ during rice reproductive growth. These tissue-specific expression patterns were highly consistent with the phenotypic variations and transcriptomic data from our previous GWAS localization analysis, leading us to hypothesize that OsARF8 and OsSTRIPE2 play pivotal roles in regulating grain width and leaf angle, spikelet length, respectively. The remaining five candidate genes also displayed clear tissue preferences: *LOC_Os09g04050* (*OsCCR1*), *LOC_Os10g37880* (*OsOG1*), and *LOC_Os06g06970* (*OsDREB1D*) were predominantly expressed in flag leaves, whereas *LOC_Os06g07030* (*OsDEAR4*) and *LOC_Os08g09190* (*OsPILS2*) were highly expressed in panicles and leaves, respectively. These expression characteristics further corroborate the phenotypic observations and transcriptomic insights from our previous GWAS work. We hypothesized that *OsDREB1D*, *OsDEAR4* and *OsPILS2* are key regulators of leaf angle and coordinate leaf angle and heading date, whereas *OsCCR1* and *OsOG1* likely mediate flag leaf photosynthetic efficiency, indirectly regulating grain development by modulating photosynthate allocation to panicles.

We systematically characterized the stress-responsive expression of the seven candidate genes using RT-PCR (**Figure 7B**). *LOC_Os08g09190* (*OsPILS2*), a key rice abiotic stress regulator, is induced by heat and cold to enhance tolerance, yet repressed under drought and salt to coordinate alternative adaptive pathways. *LOC_Os10g37880* (*OsOG1*), a constitutive physiological gene, sustains high expression under heat but is suppressed by cold and drought for resource reallocation. Under salinity, *OsOG1* is transiently activated for early signaling and later downregulated, functioning merely in the initial salt response. *LOC_Os06g07030* (*OsDEAR4*) acts as a salt-specific core gene: repressed by heat, unresponsive to cold and drought, and continuously upregulated during early-to-middle salt stress to improve salt tolerance. *LOC_Os02g41800* (*OsARF8*), a dispensable gene, is inhibited by heat and drought to redistribute resources, stable under cold, and downregulated at 24 h salt stress to maintain plant survival. *LOC_Os09g04050* (*OsCCR1*) is drought-specific; strongly suppressed by heat and cold, induced under drought and minimally expressed under salt, it exclusively mediates drought adaptation. *LOC_Os01g55974* (*OsSTRIPE2*) and *LOC_Os06g06970* (*OsDREB1D*) show no responses to heat, cold or drought. Moderately upregulated at early salt stress to initiate signaling and then restored to basal levels, they perform stage-specific functions in salt adaptation.

These findings suggest that these seven genes not only differentially regulate key rice agronomic traits but also participate in abiotic stress adaptation through distinct modes and to varying extents.

## Discussion

Mapping candidate loci or genes to predict agronomic traits is a crucial step in precision agriculture. As precision agriculture shapes the future of farming, identifying such loci or genes with predictive potential for agronomic traits is key to this agricultural revolution. This practice is not only a scientific achievement but also a vital tool for sustainable agriculture. It opens the door to a future where farmers and breeders can harness genetic insights to cultivate healthier, higher-yielding crops while conserving valuable resources. By understanding the genetic makeup of plants, producers can tailor their management strategies to maximize yields and minimize resource waste. Identifying these loci or genes is akin to finding the keys that unlock the mysteries of plant growth and development.

A key novelty of this study is the construction of predictive models. These models use seedling-stage metabolites as biomarkers to predict complex agronomic traits across multiple growth stages. Prior research has demonstrated the capacity of metabolic biomarkers for complex phenotype prediction, including metabolites associated with abiotic stress and seed quality. However, most existing work centers on yield-related heterosis. Few studies have covered a broad range of agronomic traits or combined predictive models with GWAS to identify candidate genes. Our results showed that most agronomic traits produced negative R^2^ values when modeled via LASSO and CART, revealing their poor generalization capacity. By contrast, LightGBM, RF and SWR exhibited trait-specific predictive advantages and can be prioritized according to target agronomic characteristics (**Figure 2**; **Supplementary Table 6**). These predictive models exhibited high prediction accuracy for most agronomic traits. This result aligns with the fact that yield, as a harvest-stage composite trait, is governed by complex genetic and environmental factors. Notably, predictive models maintained stable predictive ability for mature-stage agronomic traits using seedling metabolites and genetic markers. This demonstrates that seedling metabolites serve as robust proxies for these traits. Our findings offer a low-cost, time-saving strategy to predict traits at early developmental stages in rice breeding. Meanwhile, they uncover intrinsic connections between seedling metabolic profiles and late agronomic performance, and provide new insights into the continuous genetic regulation throughout rice development. (**Figures 1**, **2**; **Supplementary Table 6**). Moreover, these prediction models provide breeders with invaluable insights into prediction and precise selection of agronomic traits, acting as a trustworthy compass for breeding programs.

The genetic dissection of complex agronomic traits in rice has long been a core focus of crop genetics and breeding, with genome-wide association studies serving as a foundational tool for uncovering trait-associated genetic variants (Si et al., 2016; Yano et al., 2016; Liu et al., 2018; Zhou et al., 2020). However, traditional GWAS approaches often focus on traits or single populations, limiting their ability to capture the coordinated genetic regulation of interconnected agronomic and metabolic traits across diverse genetic backgrounds. Conventional GWAS relying solely on measured phenotypic values is highly susceptible to field environmental noise, phenotypic measurement errors, and cumulative growth-stage interference, which frequently masks minor-effect genetic loci and weakens the detection power of core regulatory variants. In contrast, performing GWAS on metabolite-predicted agronomic trait values delivers multiple unique research values that complement and breakthrough traditional analytical frameworks. Firstly, model-predicted phenotypic values mitigate random environmental fluctuations and measurement noise in field phenotyping. They effectively purify genetic signals underlying complex traits and substantially enhance the accuracy and reproducibility of QTL and SNP identification. Second, predicted phenotypes integrate early metabolic signals to uncover developmentally persistent genetic loci inaccessible to mature-stage phenotyping alone, addressing gaps in research on rice dynamic genetic regulation. Third, this approach achieves early separation of genetic effects and late phenotypes, overcoming the mature-trait restriction of traditional GWAS and supporting early molecular-assisted rice breeding. Fourth, combined with cross-population validation, predicted-trait GWAS efficiently screens conserved genetic modules across genetic backgrounds and mitigates population-specific false positives common in single-population GWAS.

In this study, we integrated GWAS of observed and metabolically predicted agronomic traits, cross-population validation, and multidimensional functional characterization to dissect the genetic architecture of key rice traits, providing novel insights into the coordinated regulation of agronomic performance and metabolic homeostasis, as well as the crosstalk between trait regulation and abiotic stress adaptation. Examining the GWAS results for the actual worth of 12 agronomic traits alongside 840 metabolites across 524 diverse rice accessions, we observed co-localization patterns among several loci. These patterns emerged not only among agronomic indicators but also between agronomic indicators and metabolites, as well as among metabolites themselves. Furthermore, we identified colocalization loci within the same trait and across different traits within the two rice accessions (**Supplementary Figures 1**, **2**; **Supplementary Tables 8**–**10**). The identification of 57 hotspots by integrating GWAS results of predicted and observed traits across two rice association populations (524 diverse accessions and 3,000 rice accessions) confirms the effectiveness of the predictive models-based approach in capturing true trait-associated QTLs. Importantly, we detected cross-population co-localized SNPs (e.g., sf0316705162) linking grain weight across populations, as well as co-localization between metabolites and agronomic traits (e.g., flavonoids and spikelet length). These findings align with the notion that metabolic pathways act as intermediaries in genotype-phenotype relationships, and conserved co-localized loci may represent core genetic modules regulating key rice traits across diverse genetic backgrounds. Such conserved loci provide valuable targets for molecular breeding, as they are likely to maintain stable effects across different environments and genetic backgrounds. Then, we identified nine hotspots significantly associated with the predictive value of 11 agronomic traits in our association panel (**Figure 3**; **Supplementary Figure 6**; **Supplementary Tables 11**–**14**). Finally, seven candidate genes identified via functional annotation were predicted to modulate yield-related and plant architecture traits in rice. These functional hypotheses represent novel inferences generated by integrating GWAS results with gene expression profiles across diverse cultivars and tissues (**Supplementary Figure 7**; **Supplementary Table 15**). Seven candidate genes by GWAS, such as *LOC_Os01g55974* (*OsSTRIPE2*), *LOC_Os02g41800* (*OsARF8*), *LOC_Os06g07030* (*OsDEAR4*), *LOC_Os06g06970* (*OsDREB1D*), *LOC_Os08g09190* (*OsPILS2*), *LOC_Os09g04050* (*OsCCR1*) and *LOC_Os10g37880* (*OsOG1*), were identified based on differential expression in different issues and haplotype analyses. However, no reports have been published on the effects of agronomic and abiotic stresses. *LOC_Os06g07030* (*OsDEAR4*) is a homolog of *Arabidopsis AtDEAR4* (*AT4G36900*), which encodes a member of the *DREB* subfamily A-5 of the *ERF/AP2* transcription factor family. It was involved in negative regulation of DNA-templated transcription and positive regulation of leaf senescence through GO analysis, but its functions related to biological functions have not been reported. Therefore, we inferred that *OsDEAR4* is most likely involved in heading stage, salt and heat stress by haplotype analysis and transcriptome analysis of different periods and tissues in rice. *LOC_Os08g09190* (*OsPILS2*) was a homolog of *Arabidopsis AtPILS2* (*AT1G71090*), which is involved in auxin homeostasis, endoplasmic reticulum-to-cytosol auxin transport, and lateral root formation (Barbez et al., 2012). It is simultaneously involved in stress such as cold, hot, salt and drought. So, we inferred that *OsPILS2* is most likely involved in heading stage and leaf angle and multiple adverse responses to stress by haplotype analysis and expression level analysis. *LOC_Os06g06970* (*OsDREB1D*) is a homologous gene of *Arabidopsis AtCBF2* (*AT4G25470*) and which encoded an *AP2/EREBP* transcription factor gene. Previous reports found that this gene was mainly involved in the response to drought, salt and cold stress in rice (Dubouzet et al., 2003; Mao et al., 2012). Therefore, based on our analysis results, we concluded that *OsDREB1D* was highly likely to participate in heading stage and leaf Angle in addition to salt stress. *LOC_Os09g04050* (*OsCCR1*) is a homologous gene of *Arabidopsis AtCCR1* (*AT1G15950*), which encoded a cinnamoyl CoA reductase and could involve in lignin biosynthetic and response to cold. Previous reports found that vessel-specific reintroduction of *CCR1* could restore vessel and xylary fiber integrity and increases biomass in dwarfed *ccr1* mutants (De Meester et al., 2017). Based on these results, we proposed that *OsCCR1* was highly likely to affect grain size, biomass, drought, heat as well as cold stress. *LOC_Os10g37880* (*OsOG1*) was a homologous gene of *Arabidopsis AT4G16765*, which encoded oxidoreductase superfamily protein. It belongs to oxoglutarate and Fe^2+^-dependent oxygenase superfamily protein, but whose biological functions have not been reported. Therefore, we concluded that *OsOG1* was highly likely to participate in grain size, cold, drought and salt stress. *LOC_Os01g55974* (*OsSTRIPE2*) was a homologous gene of *Arabidopsis AT3G48540*, and which encoded Cytidine/deoxycytidylate deaminase. Notably, the leaves of *OsSTRIPE2* mutant are streaked, with reduced chlorophyll accumulation and chloroplast distortion (Xu et al., 2014). Moreover, exogenous dUMP application partially rescued the mutant phenotype. Therefore, we inferred that *OsSTRIPE2* could play a positive regulatory role in the process of cell growth and development, especially in terms of plant height, panicle length, and salt stress. *LOC_Os02g41800* (*OsARF8*) encoded auxin response factor and is a homologous gene of *Arabidopsis AtARF16* (*AT4G30080*), which might involve in cell division, pattern specification process, response to auxin and root cap development. There were 23 and 25 *ARFs* homologous genes identified in *Arabidopsis* and rice, respectively (Wang et al., 2007). Previous reports found that most of the lines showed no obvious growth phenotype, suggesting functional redundancy among *ARFs* family proteins in *Arabidopsis*. However, *arf7* and *arf19* double mutants have a strong auxin-related phenotype, which cause severe damage to lateral roots and abnormal growth and development of hypocotyls and roots (Okushima et al., 2005). Therefore, we inferred that *OsARF8* regulates vegetative growth and development as well as responses to heat, salt and cold stress. In addition, this gene participates in reproductive development, with prominent effects on plant height and spikelet length in rice. Importantly, these genes not only regulate key agronomic traits but also participate in stress adaptation through distinct modes, suggesting that they form a multi-layered regulatory network that ensures rice survival and stable yield under complex environmental stresses. This network may underpin the trade-off between agronomic performance and stress resistance, providing a molecular basis for breeding varieties with both high yield and stress tolerance.

Despite these advances, this study has several limitations. First, the predictive models failed to accurately predict yield, likely due to the inability of seedling-stage metabolites to capture the cumulative effects of environmental factors and complex genetic regulation during the entire growth cycle, and environmental variations and inherent model limitations constrain its applicability across diverse populations. Future studies should integrate metabolites from multiple growth stages and environmental conditions to improve yield prediction accuracy. Second, although we identified candidate genes through haplotype analysis and expression profiling, further functional validation (e.g., gene knockout, overexpression, and complementation assays) is required to confirm their roles in trait regulation. Third, the regulatory interactions between the seven candidate genes remain unclear. Future studies should employ yeast two-hybrid, ChIP-seq, and transcriptomic analyses to dissect the regulatory network underlying agronomic trait coordination and stress adaptation.

Building on the limitations and novel findings of this study, future research will boost the translational value of our work. We plan to integrate multi-omics and environmental datasets to optimize the predictive model prediction framework. Functional validation and multi-omics analysis of seven candidate genes will reveal networks balancing rice yield and stress resistance.

## Conclusions

In conclusion, this study proposes an integrated strategy combining trait prediction models, cross-population GWAS and multi-dimensional functional analysis to dissect the genetic basis of rice agronomic and metabolic traits. These predictive models connect metabolic variation with macroscopic phenotypes, distinguish environmental noise from genetic variation and improve the reliability of trait quantification. Model-predicted phenotypes can act as alternative data for GWAS to improve genetic locus detection, which may facilitate the dissection of crop complex traits and molecular breeding.

## Data Availability

The original contributions presented in the study are included in the article/**Supplementary Material**. Further inquiries can be directed to the corresponding author.

## Supplementary material

Supplementary Figure 1 Colocalization loci between traditional agronomic traits in two natural population.

Supplementary Figure 2 Colocalization loci among traditional agronomic traits among metabolites in two natural population.

Supplementary Figure 3 Scatterplots between predictive value via the predictive models and manual measurement value of agronomic traits by stepwise regression model.

Supplementary Figure 4 Scatterplots between predictive value via the predictive models and manual measurement value of agronomic traits by random forest model.

Supplementary Figure 5 Scatterplots between predictive value via the predictive models and manual measurement value of agronomic traits by LightGBM model.

Supplementary Figure 6 Manhattan plots for predictive value of agronomic traits for predictive model in rice diverse accession.

Supplementary Figure 7 Summary of functional prediction of candidate genes based on GWAS and RT-PCR.

Supplementary Table 1 Description of the 524 rice accessions.

Supplementary Table 2 Description of the 3,000 rice accessions.

Supplementary Table 3 Description and abbreviation of agronomic traits.

Supplementary Table 4 Description and abbreviation of metabolic traits to 524 rice diverse accessions.

Supplementary Table 5 The data of metabolic and agronomic traits to the 524 diverse accessions.

Supplementary Table 6 Feature values by different predictive models.

Supplementary Table 7 The information of candidate SNPs of metabolic and agronomic traits were identified via GWAS to the 524 rice diverse accessions.

Supplementary Table 8 The colocalization information of candidate SNPs of different trait classes identified via GWAS to the 524 rice diverse accessions.

Supplementary Table 9 Candidate SNPs were identified via GWAS to the 3,000 rice diverse accessions.

Supplementary Table 10 The colocalization information of candidate SNPs and genes were identified via GWAS between 524 and 3,000 rice diverse accessions.

Supplementary Table 11 The information of Candidate genes through colocalization screening from 524 rice diverse accessions.

Supplementary Table 12 The colocalization information of candidate SNPs identified via GWAS among predictive value of agronomic traits, 524 and 3,000 rice diverse accessions.

Supplementary Table 13 Colocalized genes by GWAS between traditional agronomic traits and their predictive values.

Supplementary Table 14 The information of colocalized and candidate genes between traditional agronomic traits and their predictive values.

Supplementary Table 15 Expression of colocalized and candidate genes between traditional agronomic traits and their predictive values in different tissues.

Supplementary Table 16 Primers of candidate genes for RT-PCR.
